# Demystifying Failures Behind Separated Instruments: A Review

**DOI:** 10.7759/cureus.29588

**Published:** 2022-09-26

**Authors:** Manoj Chandak, Swayangprabha Sarangi, Abhilasha Dass, Monika Khubchandani, Rakhi Chandak

**Affiliations:** 1 Conservative Dentistry and Endodontics, Sharad Pawar Dental College, Datta Meghe Institute of Medical Sciences, Wardha, IND; 2 Pediatric Dentistry, Sharad Pawar Dental College, Datta Meghe Institute of Medical Sciences, Wardha, IND; 3 Oral Medicine and Radiology, Swargiya Dadasaheb Kalmegh Smruti Dental College and Hospital, Nagpur, IND

**Keywords:** techniques, root canal retreatment, biomechanical preparation, endodontics, instrument separation

## Abstract

Instrument separation is one of the most routinely encountered mishaps occurring in the arena of endodontics. Separation occurs unknowingly most times and we are left to ponder as to where to head next. It is majorly the lack of knowledge and skill that makes us panic in these times. The objective still remains to effectively clean as well as shape the root canal so that it can best receive the obturating material. Thus sudden breakage of a file during this critical phase leaves the clinician in an absolute dilemma. This review thereby throws light onto the various factors that remain in the hand of the clinician before proceeding for biomechanical preparation of the root canal so as to prevent instrument fracture. The correct application and knowledge of these techniques will prevent procedural mishaps from occurring and further enhance the quality of the treatment.

## Introduction and background

A clinician needs to recognize the underlying biological and mechanical guidelines of instrumentation in today's era of current competitive endodontics. Instrument separation in the root canal is one of the most common procedural mistakes encountered during endodontic therapy [1]. Instrument separation happens all the time, not just with students and general practitioners, but even with professional and seasoned clinicians, despite taking all precautions. With the increasing patient demands for faster treatments to be made available to them, it becomes a challenging task for the operator to satisfy the demands of the patient at the correct time as well as deliver the highest quality of service. The occurrence of procedural iatrogenic errors is inevitable during the biomechanical root canal preparation procedures. Ledging, zipping, perforation, and apical transportation of the canal along with instrument separation are some of the most commonly encountered endodontic errors encountered in routine practice [2,3]. However, this situation of instrument separation and file breakage becomes a problematic incident for the operator to ameliorate. 

Such errors reduce the effectiveness of removing the clogged debris from the canal further reducing the healing of the periapical lesions when fractured fragments are left in the canal [4]. A thorough pre-evaluation of each case is a mandatory procedure that should not be disregarded by the clinicians. Negotiation of broken instruments when they get fractured in extremely thin and highly curved root canals becomes a challenge [3-6]. The dilemma of whether the separated instrument should be left inside the canal or bypassed is always a junction where the clinician needs to rely on his clinical skills and expertise to retrieve or leave the fragment in situ. The incidences of fracture of rotary nickel-titanium (NiTi) files and conventional hand files are also shed light upon in this article, which gives a brief insight into the prime factors and the possible causes of the separation of instruments when they are mishandled wrongly within the root canal systems.

## Review

Why does instrument separation occur?

The root canal system's anatomical diversity is one of the most challenging hurdles to conquer during endodontic treatment. It is also one of the most common reasons for tool fracture [1,2]. Furthermore, these mishaps are linked to a lack of professional knowledge of the technique, excessive instrument use, insufficient use, and the amount of instrument sterilization undergone by the instrument. Overuse, or when instruments are used to their maximum limits of cycle fatigue and torsional stress, is the most prevalent cause of instrument separation or breakage. When the instrument does not bind in the canal but rotates freely in a curve while subjected to repeated tension and compression cycles, the structure disintegrates and eventually fractures.

On the contrary, torsional stress is the shear stress induced on a transverse cross-section by twisting an instrument's shaft. This occurs when the instrument's tip is jammed in the canal while the shaft is in motion or continues to rotate. Torsional stress has been found in 55.7% of the cracked files studied by Sattapan et al., while cyclic fatigue was found in 44.3% [3]. According to Pruett et al., the instrument size, radius, and angle of curvature play a role in cyclic fatigue [4]. The instrument's rotational speed has also been found to contribute to cycle fatigue. The time it takes for a file to fail falls dramatically as the rotational speed increases. It is essential to mention a multitude of other factors that shape the instrument separation. The operator's aptitude and ability are one of them. The operator's expertise and training efficiency are exceptionally crucial in clinical endodontics. For proper instrument and usage practices, several guidelines have been proposed. Before depositing files into the canal, they must be meticulously evaluated to ensure that their cutting blades are appropriately aligned. Instruments should never be put into dry or lubricated canals. Excessive force should also be avoided when handling files, and the manufacturer's specifications should be followed. Before performing on the patient, proper preclinical training and file handling should be mastered. Such handling practices would provide a realistic picture of the actual canal anatomy, eliminating file separations.

Correlation between root canal anatomy and file breakage

A variety of factors play the role of an adjunct when it comes to the breakage of files within the confines of the root canal. The major causes are as follows:

Influence of Canal Curvature Angle and Radius on File Breakage 

The likelihood of file separation is higher in the apical area of the root canal than in the middle or coronal portions. The cause is an increase in canal curvature, which causes the canal to narrow in this area. As the cyclic fatigue of the instrument grows, pronounced canal curvature reduces the life expectancy of files. The radius of canal curvature, on the other hand, is inversely related to the degree of instrument separation. The narrower the radius of curvature, the lower is the probability of file breakage [4].

The Type of Metal Alloy Used

NiTi files' extreme flexibility makes them ideal for expanding apical parts of canals with severe apical curvatures. NiTi can be pre-curved to fit effectively into canals, making them more liable to stress and strain [5].

Instrument Shape and Size Configuration

The cutting efficiency of a file is determined by the amount of file that contacts dentinal walls once it is inserted in the canal. This, on the contrary, increases friction and shortens the life of a file. The number of times a rotary file should be used is determined by its size. The force required to unwind or fracture the file increases as the diameter of the file grows [6]. Clinically significant instruments, on the other hand, should be reused with caution or thrown after multiple uses. Instruments with a rhomboid-shaped cross-sectional design are less resistant to bending forces than square-sectioned instruments [7,8].

Frequency of Instrument Usage Inside the Root Canal System

The frequency with which a file is used impacts separation because there is not enough research on how many times a file should be inserted inside the canal. However, there is enough proof that ProTaper files can be used up to ten times in simulated canals [9]. It is obvious that the instrumentation used in simulated models differs significantly from that used in extracted teeth or patients. However, it is found that a single use of a file delivers absolute fracture shielding. Endodontic files typically permanently fracture not because of how often they are used but because of how they are utilized and handled [10].

Disadvantages of instrument separation

The outcome of the cleaning and shaping process determines the success of root canal treatment. Broken files, on the other hand, are dangerous to treatment failure since their presence obstructs the cleaning, shaping, and filling processes. A detached instrument does not always imply surgery or tooth loss. When an instrument separates into the root canal, two major difficulties must be addressed to get the best possible treatment outcome. The first is the presence of a metal particle inside the tooth, which could lead to corrosion. Silver points are prone to corrosion, whereas stainless steel and NiTi rotary instruments are naturally inert. The second point of worry is that because a separated instrument prevents or hampers access to the apical foramen, the goal of root canal treatment, which is to clean and shape the canals, is jeopardized, and the treatment outcome is harmed. When fragments of broken tools impede the root canal, the case's prognosis is poor because the cleaning and disinfection of the occluded root canal may be impaired [11]. Professional expertise and ability are required for the removal of shattered instruments. Furthermore, various factors must be addressed, such as access to the fractured file, fragment size, instrument specification, prior case diagnosis, residual dental structure, and the patient's agreement after being informed of the procedure's risks and benefits [12,13].

Breakage percentages of instruments

Endodontic instrument fracture during root canal therapy is an issue every endodontist must address. Separation rates for stainless steel (SS) instruments range from 0.25 percent to six percent, while NiTi rotary instruments have rates ranging from 1.3% to 10.0%. To avoid fracture of SS instruments, they should be discarded whenever they show the slightest evidence of metal fatigue. Nevertheless, NiTi instrument separation can occur even when there are no signs of fatigue. The rotational NiTi tools minimize the danger of breaking to 0.9%, although they do not entirely lessen fracture risk [14].

Factors influencing the removal of detached instruments

Configuration of the root canal, including its cross-sectional shape, diameter, length, and curvature, are some factors that contribute to the effective retrieval of detached devices. Other characteristics include the fragmented fragment's composition, whether SS or NiTi, its length and location in the region of the canal like the apical third, middle third, and coronal third or beyond the apex, and the thickness of dentin, and the shallow concavity depth.

Consequences of fractured instruments

Shattered tools cause infected and unfilled areas to remain inside the canal system and require needless dentin removal during the cleaning and shaping of the root canal. Major complications encountered are the formation of ledges, where instruments used for removal may separate and further obscure treatment, perforation of root during staging platform preparation, and extrusion of the fragment apically due to undue pressure applied during recovery of separated instruments. Such iatrogenic errors could make the therapy process even more difficult. The therapeutic importance of fragmented files persisting within treated root canals has been debated. However, when shattered pieces affected teeth with necrotic pulp or periapical lesions, there was a reduction in periapical lesion healing. Only a few studies have successfully eliminated separation of endodontic files utilizing cutting-edge procedures. When fragments are positioned in the apical third or beyond the root canal curvature, success is less likely, especially when the root canal curvature is severe curves. Removing fractured instruments in high-risk cases with potential complications such as root perforation or root fracture is not recommended. Clinicians should evaluate each case individually and consider all aspects of the management of separation of endodontic files. Preventive procedures related to separation are of utmost importance. In light of this, understanding the mechanisms and factors contributing to filing fracture is essential.

Can instrument removal be defined as endodontic treatment success?

Strip perforation at the danger zone may be an immediate unfavourable effect of dentin removal [15]. Removing good dentin, particularly dentin from the peri-cervical area, reduces the strength of the root and puts it at risk of vertical root fracture [16-19]. Every effort should be made not to harm the patient. Endodontic treatment or prevention of apical periodontitis is the purpose of endodontics. Intra-radicular infection is the most common cause of periapical lesions [20]. A damaged file does not cause inflammation on its own [21]. It is frequently observed that teeth with shattered instruments treated many years ago still do not show any symptoms of periapical inflammation on clinical or radiologic examination. During endodontic therapy, periapical healing occurs when the microbial burden is reduced below a certain threshold. Total canal sterilization is still a pipe dream today [22]. If a fragmented instrument limits proper cleaning and the apical lesion shows no healing or a new lesion develops, as a result, apical surgery can be used without sacrificing peri-cervical dentin.

Fragment location can influence decisions in many ways

**Figure 1:**
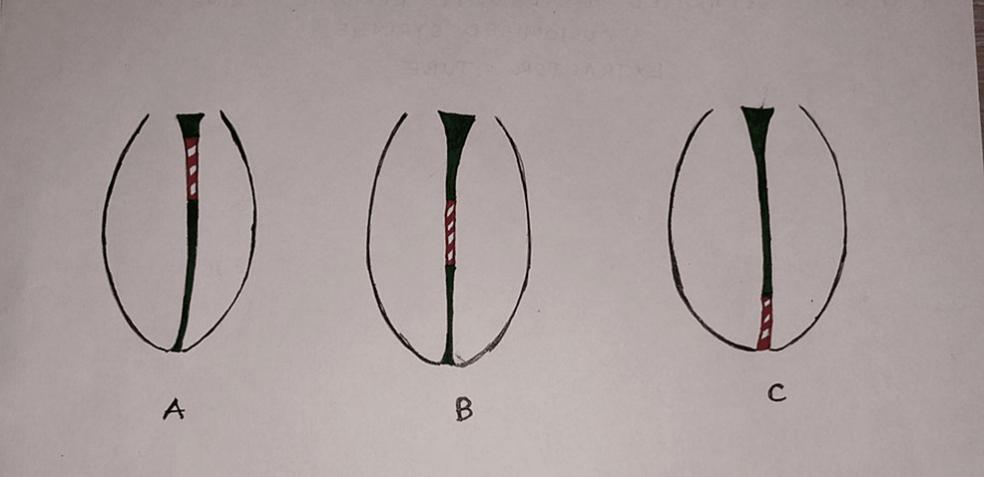
(Figure 1A) Instrument separation in coronal part of root canal, (Figure 1B) Instrument separation in middle part of root canal, (Figure 1C) Instrument separation in apical part of root canal. Image credit: Author Swayangprabha Sarangi

If fracture of instrument occurs in the coronal part of the canal, every attempt is made to remove the separated fragment by minimizing dentin removal. A wide range of grasping instruments can be put to use for this (Figure 1). If fracture of instrument occurs in the middle part of the canal, it requires the need to bypass it. If bypass cannot be done, then obturation of the canal should be done till the instrument. Follow-up is an essential requisite, and apical surgery should be undertaken in case of post-treatment endodontic disease (Figure 1). If fracture of instrument occurs in the apical part of the canal, no attempt is made to remove the fractured fragment from this region. Working length can be changed, and canal prepared up to the separated instrument location. Sodium hypochlorite agitation must be done, and obturation of the canal to be done in the same visit (Figure 1).

Separated instrument removal techniques

**Figure 2:**
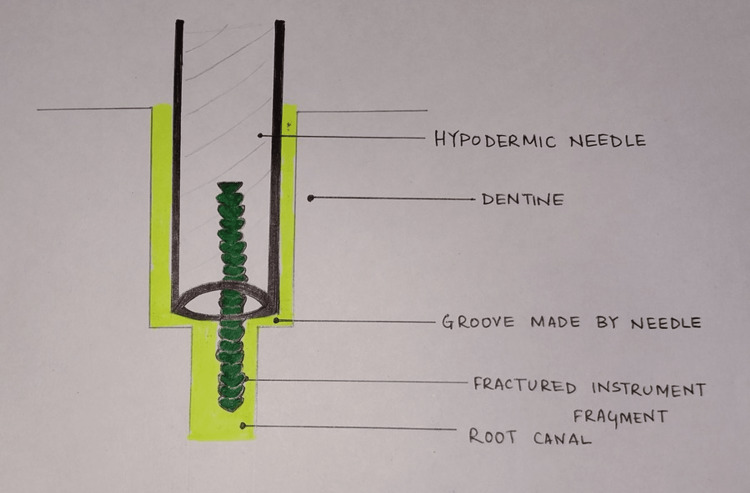
Separated instrument removal using a customized syringe extractor tube. Image credit: Author Swayangprabha Sarangi

An extensive range of techniques and instruments are used to retrieve instruments from the root canal. These enlisted are as follows: mini forceps like Stieglitz, peet silver point forceps, or endo-micro forceps, braiding technique of endodontic files using K files or H files, wire loop method using a cut disposable needle and an orthodontic wire, using a barbed broach and cotton, hypodermic surgical needles, Masserann kit instruments, extractors, ultrasonics, canal finder system, file retrieval system, softened gutta percha method, and electrochemical file dissolution technique (Figure 2).

What will happen if fractured fragment is left in-situ?

The use of bypass is strongly advisable. The canal cross-section influences this method. Slender, elongated, oval, and flattened canals and isthmuses permit the procedure, but stout canals obstruct it. If the bypass procedure was efficacious, then canal should be shaped with hand files up to size number 30, and it is then not essential to remove the fragment. Mid-appointment treatment with calcium hydroxide for two to four weeks, along with 5.25% of sodium hypochlorite agitation, is indicated for sterilization if the bypass failed. Follow-up is required after complete obturation, and apical surgery should be considered if the endodontic disease develops after therapy.

The patient should be referred to an expert or specialist if a detached instrument cannot be removed or circumvented. Cleaning and shaping up to the level of the separate piece is an alternative treatment option. It is frequently used in cases where the root canal preparation is nearing completion or when the fragment is positioned past the curve in the apical part [23-25]. Retaining the shattered instruments causes worry in the patient since it can be interpreted as a treatment failure or even clinical carelessness. It is sometimes viewed as the root of the patient's future problems. These patients are kept on follow-up regularly.

## Conclusions

The restrictions of the root canal accommodating the separated fragment, the stage of canal instrumentation at which the instrument armamentarium is available, possible complications, the strategic importance of the tooth involved, and the presence or absence of periapical pathosis are all considered when making a balanced decision. Understanding the influencing factors and the ability to make sound decision is essential. As a result, preventive strategies include case selection, clinician expertise, limited reuse, and retrieval techniques for fractured instruments. By preceding sound peri-cervical dentin, which can mainly lead to perforations and dispose the tooth to vertical root fracture, efficacious removal of broken instruments may jeopardize the tooth's long-standing result. During clinical decision-making, the doctor should consider microbiological and biomechanical factors.
